# Ajulemic acid exerts potent anti-fibrotic effect during the fibrogenic phase of bleomycin lung

**DOI:** 10.1186/s12931-016-0373-0

**Published:** 2016-05-06

**Authors:** Monica Lucattelli, Silvia Fineschi, Enrico Selvi, Estrella Garcia Gonzalez, Barbara Bartalesi, Giovanna De Cunto, Sauro Lorenzini, Mauro Galeazzi, Giuseppe Lungarella

**Affiliations:** Department of Molecular and Developmental Medicine, University of Siena, Siena, Via Aldo Moro 2, 53100 Siena, Italy; and Rheumatology Unit, University of Siena, Siena, Italy

**Keywords:** Tetrahydrocannabinol synthetic analogue, Animal model, Lung fibrosis, Anti-fibrogenic effect, PPAR-γ

## Abstract

**Background:**

Ajulemic acid (AjA) is a synthetic analogue of tetrahydrocannabinol that can prevent and limit progression of skin fibrosis in experimental systemic sclerosis. In this study we investigated whether AjA also prevents and modulates lung fibrosis induced by bleomycin (BLM) when administered in mice during the inflammatory or the fibrogenic phase of the model.

**Methods:**

The anti-inflammatory and antifibrotic efficacy of AjA was evaluated in DBA/2 mice treated orally once a day starting either at day 0 (preventive treatment) or at day 8 (therapeutic treatment) after a single intratracheal instillation of BLM. AjA was given at a dose of 1 mg/kg or 5 mg/kg. Mice were sacrificed at day 8, 14 and 21 after BLM and lungs were processed for histology and morphometry, and examined for HO-proline content and for the expression of transforming growth factor beta 1 (TGF-β1), phosphorylated Smad2/3 (pSMAD2/3), connective tissue growth factor (CTGF), alpha-smooth muscle actin (α-SMA) and peroxisome proliferator-activated receptor-gamma (PPAR-γ).

**Results:**

In the 1st week after BLM challenge, an acute inflammation characterized by neutrophil and macrophage accumulation was the main change present in lung parenchyma. The “switch” between inflammation and fibrosis occurs between day 8 and 14 after BLM instillation and involves the bronchi and vasculature. In the subsequent week (at day 21 after BLM instillation) bronchiolocentric fibrosis with significant increase of tissue collagen develops. The fibrotic response evaluated by morphometry and quantified as HO-proline in lung tissue at day 21 after BLM treatment was significantly reduced in mice receiving either AjA in the inflammatory or in early fibrogenic phase. AjA induces marked change in the expression pattern of products implicated in fibrogenesis, such as TGF-β1, pSMAD2/3, CTGF and α-SMA. In addition, AjA increases significantly the number of PPAR-γ positive cells and its nuclear localization.

**Conclusions:**

AjA treatment, starting either at day 0 or at day 8 after BLM challenge, counteracts the progression of pulmonary fibrosis. The anti-fibrotic effectiveness of AjA is irrespective of timing of compound administration. Further clinical studies are necessary to establish whether AjA may represent a new therapeutic option for treating fibrotic lung diseases.

## Background

Pulmonary fibrosis (PF) is a progressive disorder characterized by the disruption of the normal tissue architecture and function of the lung. Its main feature is the excessive synthesis and deposition of extracellular matrix in the distal airspace [1]. PF may be a fatal complication of chemotherapy and thoracic radiation. Five to 40 % of cancer patients develop drug-induced pulmonary injury, inflammation and fibrosis, resulting in significant morbidity. Mortality rates range from 2 to 80 % of cases, depending on the causal agent [2]. The probability to trigger a form of iatrogenic PF rises with cumulative doses of drugs or radiation and limits the use of otherwise effective therapies [3, 4]. Although some forms of lung fibrosis associated with some conditions (such as, desquamative interstitial pneumonia, bronchiolitis obliterans organizing pneumonia, BOOP, sarcoidosis etc.) can be effectively treated with steroids (1), other forms of PF including Idiopathic Pulmonary Fibrosis (IPF) or chemo- and radiotherapy-induced PF are usually steroid-insensitive. In the latter forms of PF and in particular in IPF, current therapies only relieve symptoms and do not alter the course of the disease [1, 5, 6]. Possibly because of the complex nature of lung fibrosis and in particular of the mechanisms underlying the development of IPF, patients respond poorly to available anti-fibrotic drugs [7]. Hence, there is an unfulfilled need for effective anti-fibrotic drugs either to treat IPF or to employ in association with cancer therapies in order to increase drug dosage and lower the risk of lung toxicity.

Different models of PF have been developed over the years to investigate the potential therapies for IPF, but unfortunately most of them mimic some, but never all features of human IPF, especially the progressive and irreversible nature of the condition. The bleomycin (BLM) model in rodents is the most commonly used.

BLM is a glycopeptide antibiotic [8] with potent anti-tumor activity against a wide range of tumors [9]. However, the therapeutic efficacy of BLM is limited by its side-effects, which include PF [10, 11].

In rodents, intratracheal administration of BLM induces acute alveolitis and interstitial inflammation, which are characterized by the recruitment of leukocytes within 1 week [12]. The “switch” between inflammation and fibrosis occurs between day 8 and 14 after BLM instillation and the fibrotic response is characterised by fibroblast proliferation and synthesis of extracellular matrix [13]. Various types of resident cells, including macrophages, fibroblasts, endothelial and epithelial cells, or migrating cells such as neutrophils and fibrocytes, have been implicated in the development of PF [14].

Over the years, numerous agents have been shown to inhibit fibrosis in BLM model. However, to date none of these compounds are used in the clinical management of IPF and none has shown a comparable anti-fibrotic efficacy in humans. The most part of these compounds was tested in BLM model “in a preventive manner” during the first 7 days after BLM administration when, an interference with the inflammatory response can modify or abolish the following fibrogenic phase. Actually, in human IPF the inflammation phase is not recognized and patients come to the attention of the physician only in the advanced fibrogenic phase of the disease.

Only few drugs have been shown to reduce fibrosis when used during the fibrogenic phase of BLM model but unfortunately most of them were abandoned after clinical trials for lack of benefit or for their toxicity [for a review see 15].

In previous studies we demonstrated that some synthetic cannabinoids are effective in preventing dermal collagen accumulation in a model of systemic sclerosis [16, 17]. In particular, ajulemic acid (AjA), a no-toxic synthetic analogue of tetrahydrocannabinol, exerts potent anti-fibrotic effects in experimental models of systemic sclerosis.

Given the necessity to discover new drugs for the therapy of human pulmonary fibrosis we decided to evaluate the potential anti-fibrotic effect of AjA either in the early period of BLM-induced inflammation or in the fibrogenic phase in order to assess its real anti-fibrotic property.

## Methods

### Animals

Male DBA/2 mice (Charles River, Calco, Italy) of 6–8 weeks of age were used in this study. Mice were group-housed in individual cages in climate-controlled animal quarters and given water and food (Mucedola Global Diet 2018, Harlan, Italy) *ad libitum*, while a 12-h on 12-h off light cycle was maintained. All protocols were approved by the University of Siena Committee for Animal Experimentation - OBA (Project G 2207/09 #6; G 2007/11 #2). The animal experimentation was conducted in conformity with the “Guiding Principles for Research involving Animals and Human Beings”.

### Experimental design

**Fig. 1:**
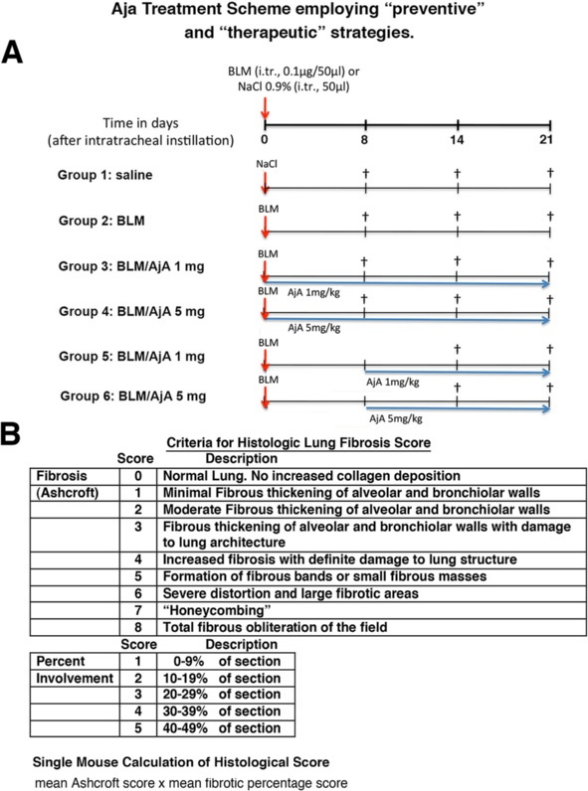
Scheme of Treatment and Criteria for Histologic Fibrosis Scores. **a** “Preventive” and “Therapeutic” Animal Treatment with Ajulemic Acid (AjA) in Bleomycin (BLM) Model. **b** Histologic Lung Fibrosis Score was determined in individual animals by multiplying Ashcroft Score by the mean of extent of fibrosis scored from 1 to 5 as reported in figure

Saline and BLM groups received a single intratracheal (i.tr.) instillation of 50 μl of saline solution (control saline) or 0.1 μg of BLM (Sanofi-Aventis S.p.A, Milan) in 50 μl of saline solution. We defined the day of BLM administration as day 0, allowing the association of this time point with the schedule of compound administration. Six groups of 14 animals each were used in this study to evaluate the efficacy of AjA (Corbus Pharmaceuticals, Norwood, MA, USA) by starting the treatment in the inflammatory (at day 0) or in early fibrogenic phase (at day 8 after BLM application) (Fig. 1a). This methodological approach was chosen in order to evaluate whether AjA treatment is able to interfere with the inflammatory response and/or with the fibrogenic changes caused by BLM instillation.

In the “preventive protocol of treatment”, experimental groups received *per os* either 1 mg or 5 mg/Kg of AjA in 100 μl safflower oil, at 1 h before BLM treatment and then once a day for the following 8, 14 and 21 days. In the “therapeutic protocol of treatment”, additional two groups of animals received daily *per os* AjA at a dose of 1 mg and 5 mg/Kg, respectively, starting at day 8 after BLM instillation for the following 14 and 21 days (Fig. 1a).

BLM i.tr. instillation was performed under light ether anaesthesia as reported in details in previous papers [18, 19].

At the selected time points (Fig. 1a), the animals were injected with an overdose of pentobarbital sodium. The lungs were then excised and processed for histological and biochemical analysis.

To exclude the interference of safflower oil (used as AjA vehicle) in our study, we carried out preliminary experiments by treating *per os* animals with safflower oil. Briefly, in this preliminary experiment, 5 mice/group were treated *per os* with 100 μl safflower oil 1 h before instillation with saline or BLM, and then once a day for the following 14 and 21 days. At day 14 and 21, mice were sacrificed and lungs were analysed by morphology. No changes were detected in mice treated with safflower oil alone before saline administration. Additionally, no appreciable morphological differences were observed between groups receiving safflower oil plus BLM or BLM alone.

### Histology and morphometry

The lungs of seven mice from different groups of treatment were fixed i.tr. with buffered formalin (5 %) at a constant pressure of 20 cm H_2_O at least for 24 h. Lungs were fixed, dehydrated, cleared in toluene, and embedded in paraffin.

### Histopathological assessment of lung fibrosis

Lungs of seven animals from each experimental groups excised 21 days after BLM or saline treatment were used for the histopathological assessment of fibrosis.

Three systems were used to assess fibrosis on Haematoxylin & Eosin and Masson’s trichrome stained lung sections (5 μm thick) (see Fig. 1b) [20, 21]. The first scoring system was used to determine the extent of the fibrotic areas by calculating the percentage of the total lung surface involved. The second scoring system was used to evaluate the severity of the lesions. This was achieved by using the Ashcroft scoring system adapted by Theiss [20, 21] (see Fig. 1b). Finally, we determined the mean histological score by taking into account both the severity (Ashcroft score) and the extent of the fibrotic lesions [22], which was scored from 1 to 5 on the basis of the mean percentage of fibrotic areas involved in individual animals. This was assessed for each animal, and multiplied by the mean Ashcroft score (Fig. 1b).

The extent of fibrotic areas was carried out on Masson's trichrome stained slides at final magnification of x100–200. For stereological determination, randomly sampled areas of lungs were analyzed using stereology grid-counting techniques to quantify areas of fibrotic disease.

### Immunohistochemistry

Tissue sections (5 μm thick) from the different groups of mice were stained for transforming growth factor (TGF)-ß1, connective tissue growth factor (CTGF), alpha-smooth muscle actin (α-SMA), phosphorylated Smad2/3 (pSMAD2/3) and peroxisome proliferator-activated receptor gamma (PPARγ).

The sections were pre-treated with 3 % hydrogen peroxide for blocking the endogenous peroxidase. On PPARγ sections, no hydrogen peroxide pre-treatment was performed. Antigen retrieval was performed for the immunodetection of TGF-ß1, pSMAD2/3 and PPARγ by heating the sections in a microwave for 20 min in 0.01 M pH 6.0 citrate buffer and allowing to cool slowly to room temperature (RT). All the sections were incubated with 3 % bovine serum albumin for 30 min at room temperature to block non-specific antibody binding.

Sections were incubated overnight at 4 °C in the primary antibody: rabbit Ab to mouse TGF-ß1 (Insight Biotechnology LTD, Wembley, UK) diluted 1:20; rabbit Ab to mouse CTGF (Abcam, Cambridge, UK) diluted 1:200; rabbit Ab to mouse pSMAD2/3 (Millipore, Merck KGaA, Darmstadt, Germany) diluted 1:100; rabbit Ab to mouse PPARγ (Cell Signaling Technology, Beverly, MA) diluted 1:100.

After rinsing with PBS, TGF-ß1 and pSMAD2/3 slides were incubated with sheep anti-rabbit IgG (1:200) for 30 min at RT followed by incubation with peroxidase-antiperoxidase complex, prepared from rabbit serum.

CTGF sections were incubated with the appropriate biotin-conjugated secondary antibody and subsequently with streptavidin/peroxidase solution.

Colour development was performed using 3,3′-diaminobenzidine tetra hydrochloride as a chromogen (DAB; Vector Laboratories, Burlingame, CA).

The sections for PPARγ were rinsed in PBS and incubated with goat polyclonal anti-rabbit biotinylated IgG (1:200) (Vector Labs, Burlingame, CA) for 30 min. at room temperature. The staining was revealed by adding Streptavidin-alkaline phosphatase (BD Pharmingen, Buccinasco, Italy).

After rinsing in 0.01 M PBS containing 0.1 % Triton X-100, the alkaline phosphatase reaction was developed with NBT/BCIP stock solution (Roche Diagnostics, Milan, Italy) as chromogen diluted in 0.1 M TRIS buffer, pH 9.5, 0.05 M MgCl_2_, 0.1 M NaCl, 2 mM levamisole.

α-SMA protein was immunohistochemically evaluated on paraffin sections using mouse monoclonal α-SMA Ab (Sigma, St. Louis, MO, USA) diluted 1:200. Reaction was visualized using the M.O.M. immunodetection kit (Vector Laboratories, Burlingame, CA) and DAB as substrate. The Vector M.O.M. immunodetection kit is designed specifically to localize mouse primary monoclonal and polyclonal antibodies on mouse tissues by using a novel blocking agent and reducing undesired background staining.

As negative controls, all primary antibodies were replaced by non-immunised specific serum.

Immunohistological score for pSMAD2/3, CTGF and PPAR-γ was carried out at x200 magnification. The number of positive cells in lung sections (10 random microscopic fields per lung section in 5 different sections) was counted manually in a blinded manner and averaged. Cells that showed nuclear PPAR-γ positivity were enumerated.

The extent of α-SMA positive areas was carried out on immunostained slides at x200 final magnification. For stereological determination randomly sampled areas of lungs were analyzed using stereology grid-counting techniques to quantify positive areas.

### Hydroxyproline assay

To quantify lung collagen at the different time points, 7 animals from different groups were used. Mice were anaesthetized with sodium pentobarbital and sacrificed by severing the abdominal aorta. After thoracotomy, the thoracic viscera were immediately removed. The lungs were weighed and homogenized (1:9, weight: volume (w: v). An aliquot was hydrolysed in 6 N HCl and the remaining part was used for Western Blot analysis. Hydroxyproline (HO-proline) was determined in lung hydrolysates according to the method of Kivirikko et al. [23, 24] and data were expressed as μg · lung^−1^.

### ELISA assay

Quantification of TGF-β1 levels in tissue lung was measured by ELISA following the manufacturer’s protocol (R&D Systems, Minneapolis, MN).

### Western blotting

Western blotting was used to determine α-SMA expression in pulmonary tissues from 7 animals of the different groups. The protein concentration of each sample was assayed using Bio-Rad Bradford protein assay. Fifty micrograms of lung homogenate were electrophoresed through a SDS-polyacrylamide gel and transferred to a nitrocellulose membrane. After blocking with 5 % non-fat powdered milk in Tris-buffered saline/Tween 20, the blots were incubated with a mouse monoclonal anti-α-SMA Ab (1:8000; Sigma, St. Louis, MO, USA) at 4 °C overnight. After washing with Tris-buffered saline/Tween 20, they were incubated with a secondary anti-mouse conjugated to horseradish peroxidase (1:10000) (Roche Molecular Biochemicals, Milan, Italy). The blot was developed using the enhanced chemiluminescence method ECL-Kit Lumi-LightPlus (Roche Molecular Biochemicals, Milan, Italy) according to the manufacturer’s instructions. The membranes were re-probed with rabbit polyclonal to glyceraldehyde 3phosphate dehydrogenase (GAPDH) (1:2500) (Abcam, Cambridge, UK) to normalize for protein loading. Each band was scanned by densitometry analysis and normalized to GAPDH.

### Statistical analysis

The significance of the differences was calculated using one-way analysis of variance. A p value of less than 0.05 was considered significant.

## Results

To test the hypothesis that AjA administration prevents the development of pulmonary fibrosis, we used the single-dose model of i.tr. BLM instillation in mice [25] and started AjA treatments at the same day of BLM administration (Fig. 1a, Groups 3 and 4) or at day 8 during the fibrogenic phase (Fig. 1a, Groups 5 and 6).

### AjA treatment limits the development of pulmonary fibrosis when used in “preventive manner”

**Fig. 2:**
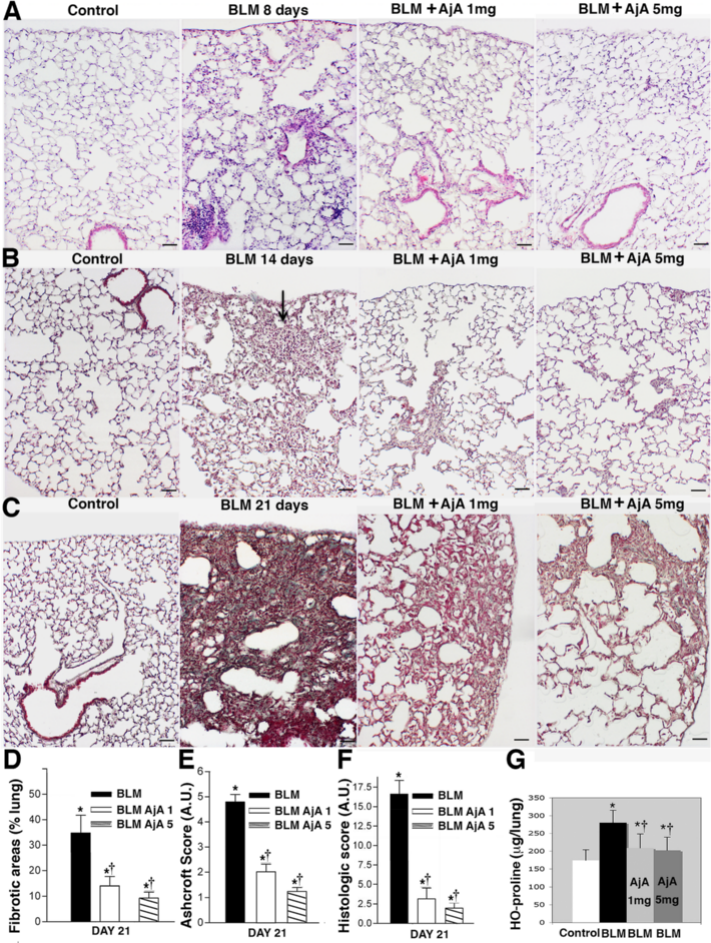
AjA reduces inflammation and provisional matrix deposition in BLM-lung and prevents the development of lung fibrosis when administered in preventive manner in BLM treated mice. **a** Lung slides of mice from the different experimental groups at day 8 from the start of treatment are shown. Haematoxylin & Eosin staining, scale bars = 60 μm. **b** Lung slides from mice at day 14 from the start of treatment. Note the large area of inflammation with presence of “provisional” matrix (arrows) in mice treated with BLM alone. Mild changes are seen in mice treated with AjA at a dose of 1 mg/Kg and 5 mg/Kg. Masson’s trichrome staining, scale bars = 60 μm. **c** Lung slides from mice of the different experimental groups at day 21 from the start of treatment. Consolidated areas of fibrosis are evident in BLM-treated mice. Reduced areas of fibrosis are seen in groups receiving in the same day of BLM delivery AjA at a dose of 1 mg/Kg and 5 mg/Kg. Masson’s trichrome staining, scale bars = 60 μm. **d** Extent of the fibrotic areas as percent of surface area involved. Data are reported as mean ± SD. *p < 0.05 *vs* control group. † *p* < 0.05 *vs* BLM group. **e** Ashcroft score: data are reported as mean ± SD. *p < 0.05 *vs* control group. † *p* < 0.05 *vs* BLM group. **f** Histological score: data are reported as mean ± SD. *p < 0.05 *vs* control group. † *p* < 0.05 *vs* BLM group. **g** Lung HO-proline expressed as μg/lung in the various experimental groups. Data are reported as mean ± SD. *p < 0.05 *vs* control group. † *p* < 0.05 *vs* BLM group

The exposure to BLM in DBA/2 mice resulted at day 8 after treatment in the development of sub-pleural areas of inflammation that extended into the lung parenchyma and involved the bronchi and vasculature (Fig. 2, panel a). Later on, more severe areas of fibrosis with marked disruption of the alveolar unit and increased deposition of collagen were observed in BLM-treated mice.

In particular, 14 days after BLM, DBA/2 mice showed large areas of inflammation and “provisional” matrix (Fig. 2, panel b, arrow). At day 21, consolidated areas of fibrosis with collagen accumulation (“sea green areas”) were evident (Fig. 2, panel c) and involved 36.84 ± 11.47 % of the lungs (Fig. 2d).

Control lungs of saline-instilled mice showed a normal architecture (Fig. 2, panels a–c). The morphological examination of lung sections revealed that AjA administered in preventive manner (Groups 3 and 4 of Fig. 1a) markedly reduced the number of inflammatory cells at day 8 (Fig. 2, panel a) and attenuated the collagen deposition and destruction of lung architecture at day 14 (Fig. 2, panel b) and 21 (Fig. 2, panel c) as compared to mice treated with BLM alone (Fig. 2, panels a–c).

Twenty-one days after BLM administration, DBA/2 mice treated with AjA showed a marked and significant reduction of fibrotic areas (Fig. 2d) as well as of Ashcroft (Fig. 2e) and histological scores (Fig. 2f).

The effects of AjA in reducing collagen accumulation were also determined by analysing lung HO-proline content of mice after BLM exposure. The i.tr. instillation of BLM in mice resulted in a significant increase in lung HO-proline at day 14 after treatment that peaked at day 21. AjA treatment (1 mg/kg/day and 5 mg/kg/day) significantly reduced total lung HO-proline content in samples of BLM-instilled mice at day 21 after treatment (Fig. 2g).

**Fig. 3:**
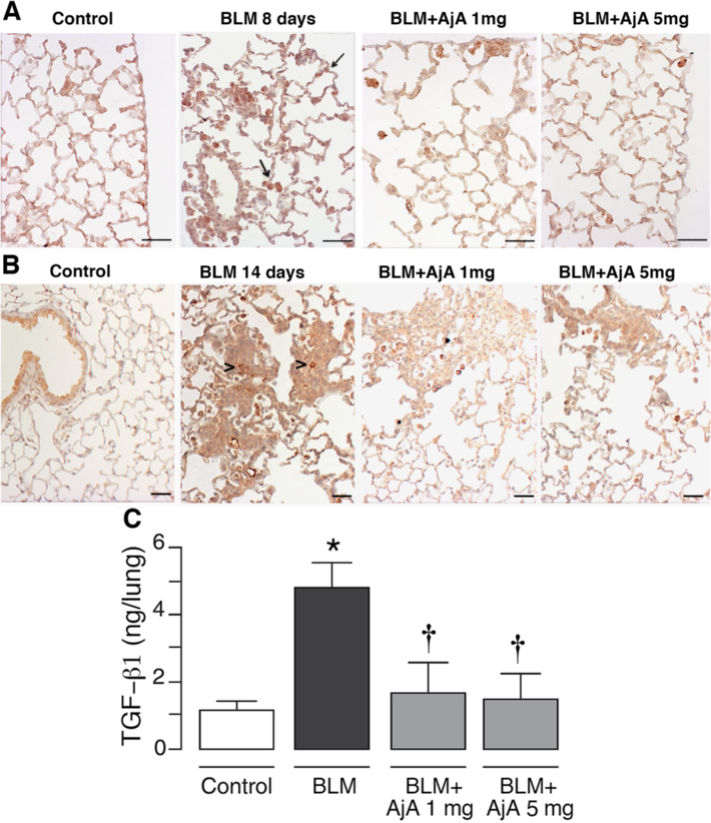
AjA treatment reduces the expression of TGF-β1 in lungs of BLM-treated mice. Representative immunohistochemical staining of TGF-β1 in the different experimental groups at day 8 **a** and 14 **b** are shown. An immunopositive reaction is observed on epithelial cells and alveolar macrophages as marked by arrows. TGF-β1 positive cells are also seen in consolidated areas of fibrosis (arrowheads). Scale bars = 60 μm. **c** Tissue levels of TGF-β1 measured by ELISA at day 14 from BLM challenge. Data are reported as mean ± SD. *p < 0.05 *vs* control group. † *p* < 0.05 *vs* BLM group

In control animals, a mild positivity of TGF-β1 was seen on some alveolar macrophages and some cells of the airways epithelium (Fig. 3, panels a and b). In BLM-instilled mice, the intensity of TGF-β1 staining (arrows) markedly increased on the cells at both day 8 and day 14 after BLM treatment (Fig. 3, panels a and b, respectively). TGF-β1 positivity was also observed on some alveolar epithelial cells and the extracellular matrix (arrowheads) present in fibrotic areas (Fig. 3, panel b). A less widespread and intense TGF-β1 reaction was seen in mice treated with AjA (1 mg/kg/day and 5 mg/kg/day) at day 8 (Fig. 3, panel a) and 14 (Fig. 3, panel b). Additionally, a significant effect of AjA in reducing TGF-β1 tissue levels was demonstrated by ELISA (Fig. 3c).

**Fig. 4:**
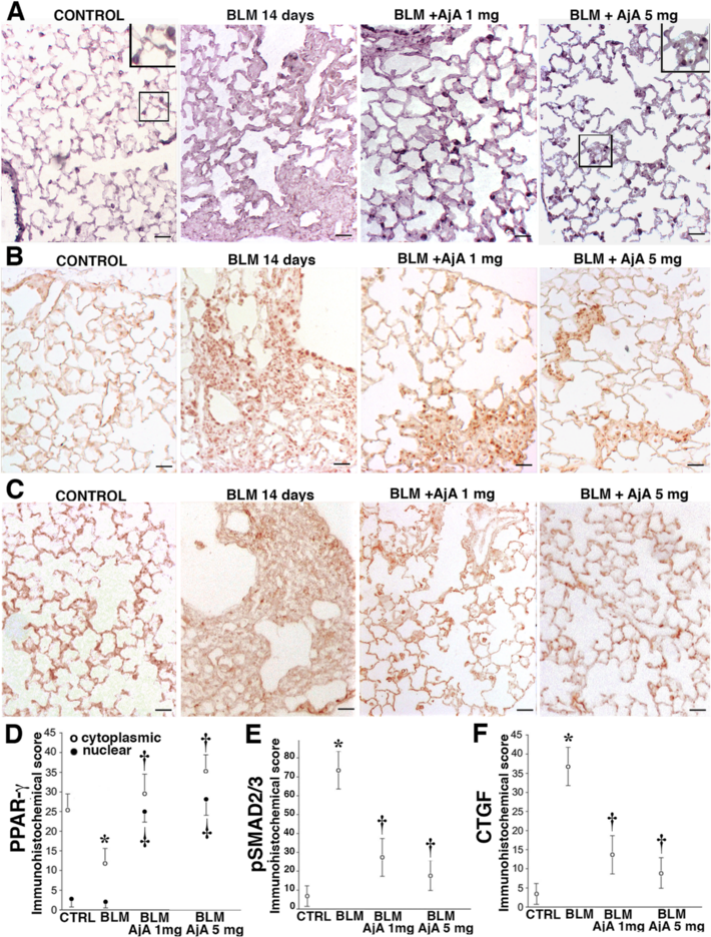
AjA treatment is followed by enhanced PPAR-γ expression, reduced pSMAD2/3 nuclear localization and reduced expression of CTGF. **a** Representative immunohistochemical staining of PPAR-γ in lung section from animals of the different experimental groups at 14 days after BLM delivery. Scale bars = 60 μm. **b** Immunohistochemical staining of pSMAD2/3 in lung section from control mouse, BLM-treated mouse and mice receiving in the same day of BLM delivery AjA at a dose of 1 mg/Kg and 5 mg/Kg, at 14 days after BLM delivery. Scale bars = 60 μm. **c** Representative immunohistochemical staining of CTGF in lung section from the different experimental groups. Scale bars = 60 μm. **d**–**f** Immunohistochemical scores relative to cytoplasmic and nuclear localization of PPAR-γ, pSMAD2/3 and CTGF positive cells from the various experimental groups analysed in the preventive study. Data are reported as mean ± SD. *p < 0.05 *vs* control group. † *p* < 0.05 *vs* BLM group

The increased expression of TGF-β1 observed in mice after BLM treatment was accompanied by a decreased expression of PPAR-γ. In lungs from control mice, PPAR-γ was detectable in several different types of cells, where it appeared to be localized in the cytoplasm (Fig. 4a, inset). After BLM treatment a progressive loss of PPAR-γ expression was observed mainly in the fibrotic areas (Fig. 4, panel a). Administration of AjA (1 mg/kg/day and 5 mg/kg/day), in BLM treated mice, markedly increased PPAR-γ positivity, with a prevalent nuclear localization (Fig. 4, a inset).

In Fig. 4d, we report the values of the immunohistochemical score of cytoplasmic and nuclear PPAR-γ staining in the various experimental groups analysed in the preventive study.

An immunohistochemical faint reaction for pSMAD2/3 was seen in lungs of control mice (Fig. 4, panel b). It is worth to note that, the enhanced nuclear expression and retention of pSMAD2/3 observed in BLM treated mice was efficiently counteracted by AjA administration. In Fig. 4e, we report the values of the immunohistochemical scores of pSMAD2/3 from the various experimental groups.

A mild reaction for the fibrogenic cytokine CTGF was appreciated in lung slices from control mice (Fig. 4, panel c). At day 14 after BLM treatment an increased amount of CTGF was found in fibrotic areas of lung tissue. CTGF was markedly reduced in tissue samples of mice treated with AjA both at the dose of 1 mg/kg/day and 5 mg/kg/day. In Fig. 4f we report the values of the immunohistochemical score of CTGF in the various experimental groups.

**Fig. 5:**
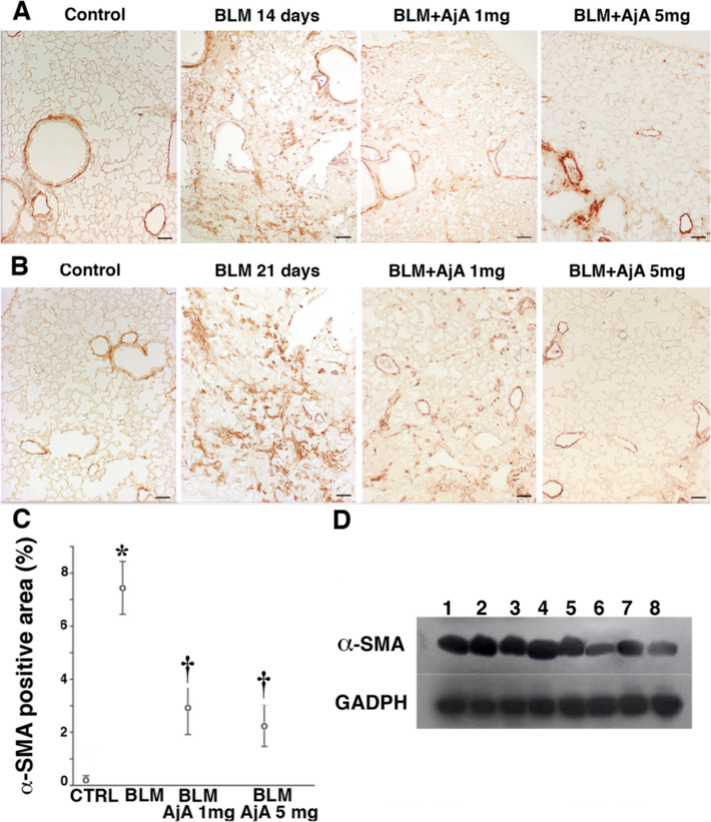
AjA treatment is followed by a reduced expression of α-SMA. Representative immunohistochemical staining of α-SMA in lung sections from animals from the different experimental groups at day 14 **a** and 21 b after BLM treatment, receiving AjA at 1 h before BLM challenge. Scale bars = 60 μm. **c** Immunohistochemical score for α-SMA in the different experimental groups. Data are reported as mean ± SD. *p < 0.05 *vs* control group. † *p* < 0.05 *vs* BLM group. **d** Representative Western blot analysis of α-SMA from total lung samples of control, BLM-instilled and AjA-treated mice at 14 and 21 days after the start of treatments. Lanes (1) and (3) = Control samples. Lanes (2) and (4) = BLM-instilled mice at 14 and 21 days. Lanes (5) and (7) = AjA 1 mg at 14 and 21 days. Lanes (6) and (8) = AjA 5 mg at 14 and 21 days

In lungs of control mice (Fig. 5, panel a and b), α-SMA was only found around arterial vessels and bronchial and bronchiolar walls. BLM treatment induced increased expression of α-SMA in mouse lungs both at day 14 and 21. This protein is a sensitive indicator of myofibroblast differentiation and thus is a very good indicator of the presence of active fibrogenic cells in lung tissue. The appearance of newly differentiated α-SMA positive cells was markedly reduced by AjA treatment both at day 14 (Fig. 5, panel a) and 21 (Fig. 5, panel b) after BLM instillation. In Figs. 5, c and d, we report the immunohistochemical scores and the Western blot analysis of α-SMA in mouse lungs from the various experimental groups used in the preventive study.

### Delayed treatment with AjA effectively limits pulmonary fibrosis progression by decreasing fibrogenesis

**Fig. 6:**
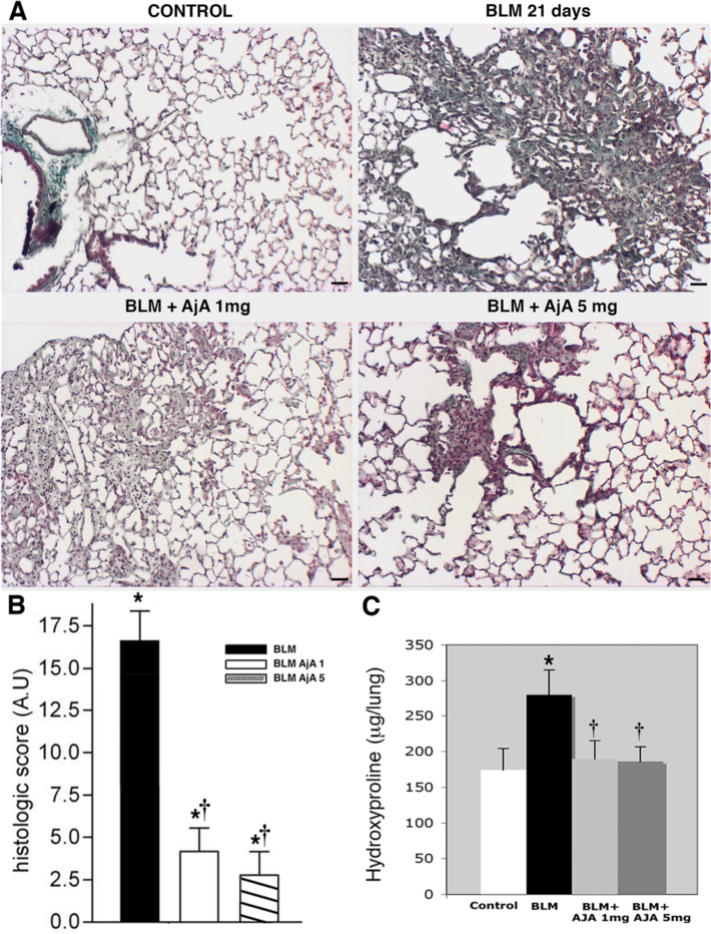
Treatment of AjA in the fibrogenic phase of BLM-lung effectively limits the development of fibrosis as revealed by lung morphology and morphometry as well as HO-proline content. **a** Lung slides from mice at day 21 from control mouse, BLM-treated mouse, and mice receiving AjA at a dose of 1 mg/Kg and 5 mg/Kg at day 8 after BLM delivery. Masson’s trichrome staining, scale bars = 60 μm. **b** Histologic score in the different mouse groups. Data are reported as mean ± SD. *p < 0.05 *vs* control group. † *p* < 0.05 *vs* BLM group. **c** Lung HO-proline expressed as μg/lung in the various experimental groups. Data are reported as mean ± SD. *p < 0.05 *vs* control group. † *p* < 0.05 *vs* BLM group

In order to test the therapeutic value of AjA in preventing fibrogenesis, animals were treated with the synthetic cannabinoid in the fibrogenic stage of BLM lung, starting at day 8 from BLM administration (Fig. 1, Groups 5 and 6). Daily treatment of mice at a dose of 1 and 5 mg/Kg markedly reduced at different extent the fibrotic reaction observed at day 21 (Fig. 6a). For comparison, representative lung histologic section from control and BLM treated mice are reported in the same panel. In Fig. 6b the histologic score of fibrosis of lungs from the various experimental groups analysed in fibrogenic study is presented. The decrease in collagen deposition by delayed AjA treatment observed in histological slides after Masson-trichrome staining (Fig. 6, panels a and b) was also confirmed by the decrease of total lung HO-proline content (Fig. 6c).

**Fig. 7:**
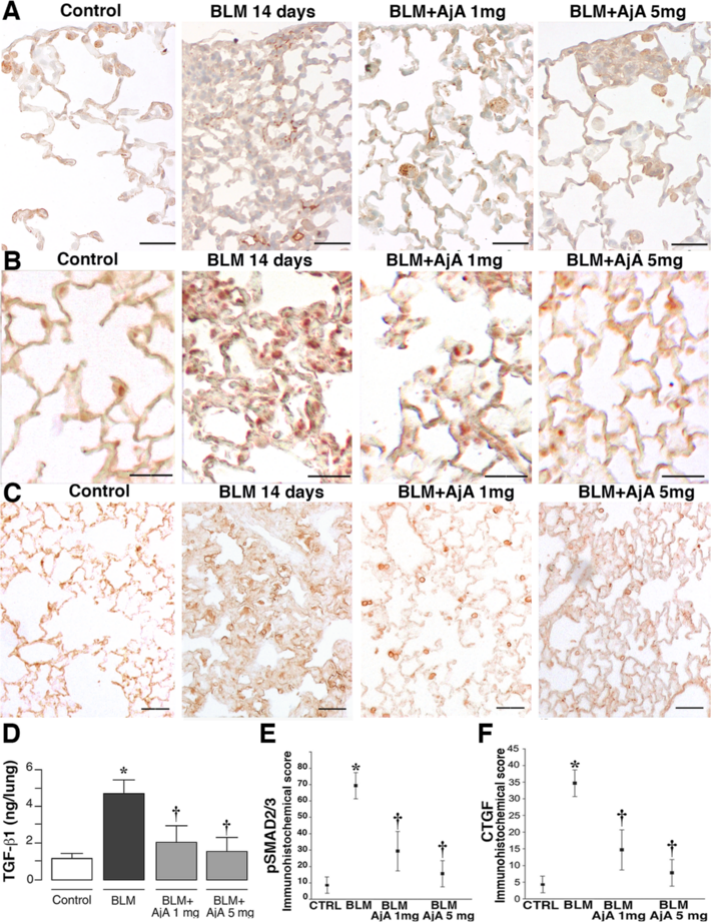
AjA treatment in the fibrogenic phase reduces TGF-β1, pSMAD2/3 and CTGF expression. **a** Immunohistochemical staining of TGF-β1 at day 14 after BLM delivery in lung sections from control mouse, BLM-treated mouse with consolidated area of fibrosis, and mice receiving at day 8 from BLM delivery AjA at a dose of 1 mg/Kg and 5 mg/Kg. Scale bars = 60 μm. **b** Imunohistochemical staining of pSMAD2/3 in lung sections from the different experimental groups at day 14. Scale bars = 60 μm. **c** Representative immunohistochemical staining of CTGF in lung sections from the different experimental groups. Scale bars = 60 μm. **d** ELISA analysis of TGF-β1. BLM increased tissue levels of TGF-β1. **e**-**f** immunohistochemical score for pSMAD2/3 and CTGF in the different experimental groups. * *p* < 0.05 vs control; † *p* < 0.05 vs BLM

Representative immuno-histochemical reactions for TGF-β1, pSMAD2/3 and CTGF detected at 14 days after BLM administration are reported in Fig. 7, panels a, b and c, respectively. The reduction of fibrosis score observed in mice treated with AjA (at a dose of 1 and 5 mg/Kg) was preceded at day 14 by a low expression (Fig. 7, panel a) and decreased tissue levels of TGF-β1 (Fig. 7d), by a low nuclear expression and reduced nuclear retention of pSMAD2/3 (Fig. 7b) and by a marked reduction of CTGF (Fig. 7c) as compared to mice treated with BLM alone. A faint reaction for these products was observed in lung tissues from control mice. The values of immunohistochemical scores for pSMAD2/3 and CTGF obtained in lungs from animals of all experimental groups are reported in Fig. 7e and f.

**Fig. 8:**
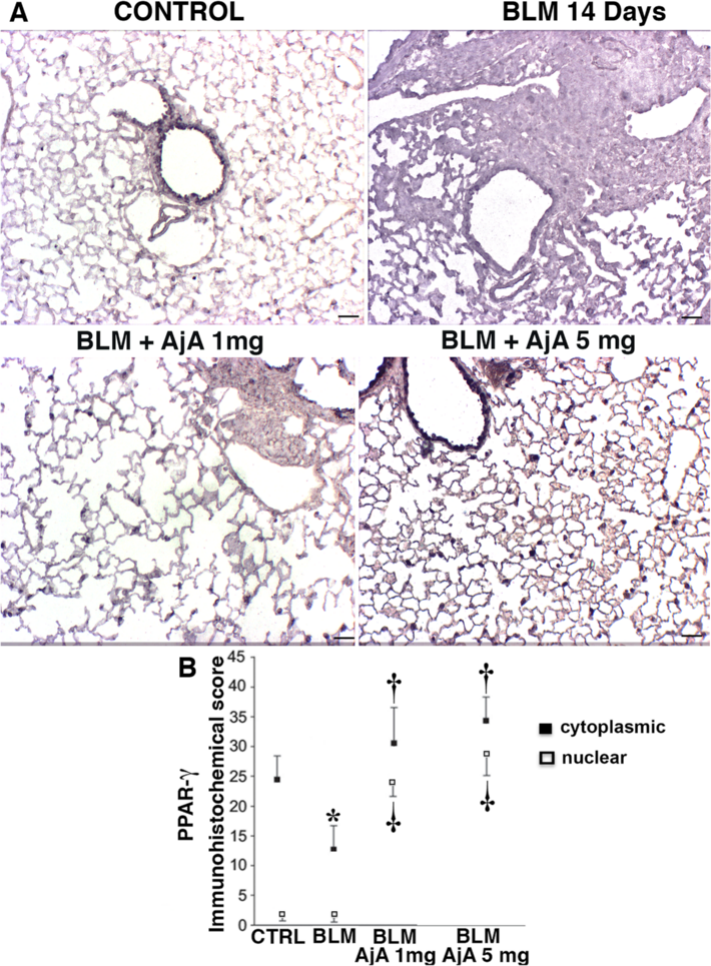
AjA treatment in the fibrogenic phase increases the number PPAR-γ positive cells as well as the nuclear localization of the receptor. **a** Representative immunohistochemical staining of PPAR-γ in lung sections from the different experimental groups at 14 day after BLM challenge. PPAR-γ positive cells were significantly decreased after BLM challenge. Additionally, the nuclear stain for this receptor was very low in lung sections of control and BLM treated mice. AjA treatments at different doses significantly increase the number of PPAR-γ positive cells and its nuclear stain in lung slides. Scale bars = 60 μm. **b** The immunohistochemical score of cytoplasmic and nuclear staining for PPAR-γ in lung tissue from the various experimental groups is shown

As previously mentioned, PPAR-γ was detectable in several different types cells in lungs from control mice, where it appeared to be localized in the cytoplasm. A decreased expression of PPAR-γ was seen in lung sections of mice after BLM challenge (Fig. 8a). Similar to what observed in the preventive study, the low grade of fibrosis detected in AjA-treated groups was accompanied by enhanced expression of PPAR-γ with an increased nuclear localization. The values of immunohistochemical scores for cytoplasmic and nuclear staining of PPAR-γ in lungs from the various experimental groups are reported in Fig. 8b.

**Fig. 9:**
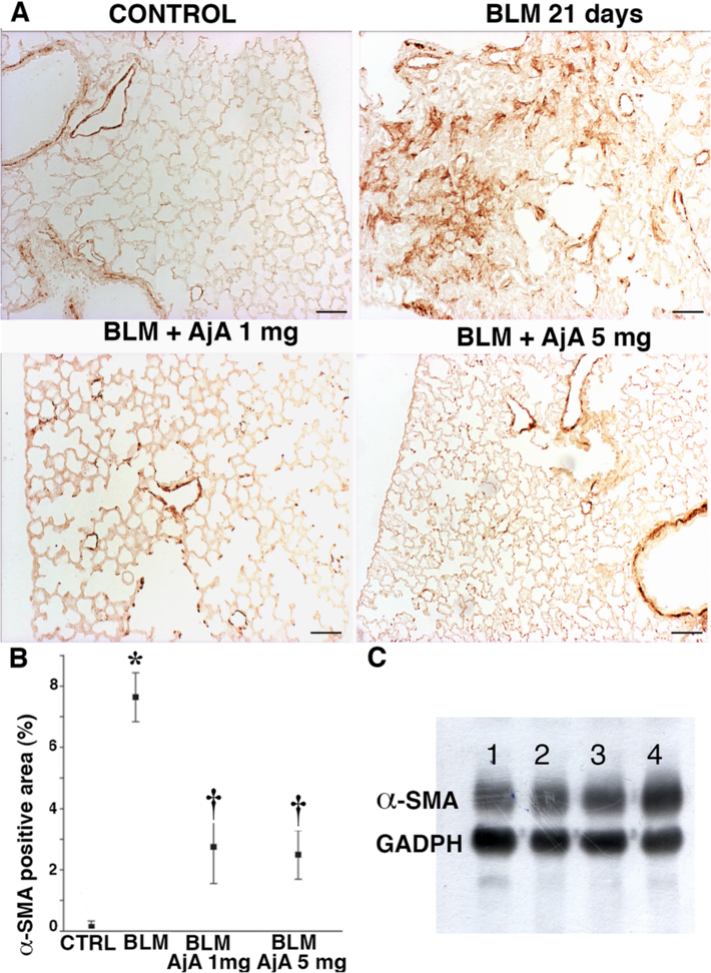
AjA treatment in the fibrogenic phase significantly decreases the expression of α-SMA in lungs of BLM treated-mice. **a** Immunohistochemical staining of α-SMA at day 21 after BLM delivery in lung section from control mouse, BLM-treated mouse with consolidated area of fibrosis, and mice receiving at day 8 from BLM delivery AjA at different doses. Scale bars = 60 μm. **b** Immunohistochemical score of α-SMA in the lungs of the different experimental groups. * *p* < 0.05 vs control; † *p* < 0.05 vs BLM. **c** Representative Western blot analysis of α-SMA from total lung samples of control, BLM-instilled and AjA-treated mice at 21 days after BLM challenge. Lane (1) = control sample. Lane (2) = BLM + AjA 5 mg. Lanes (3) = BLM + AjA 1 mg. Lanes (4) = BLM-instilled sample

Representative immunohistochemical reaction for α-SMA in lung tissue from mice of the various experimental groups is reported (Figs. 9a). A marked increase of α-SMA was seen in lung sections of BLM treated mice as compared with those of control animals at day 21. An evident reduction of α-SMA positive cells was appreciated in AjA treated groups at the different doses as compared to mice treated with BLM alone. The values of immunohistochemical score for α-SMA are shown in Fig. 9b. Similarly, as tested by western blotting a decreased tissue expression of α-SMA was observed in AjA treated groups (Fig. 9c, lanes 2 and 3) in respect to mice treated with BLM alone (Fig. 9c, lane 4). In the same figure, α-SMA from control group is shown in lane 1.

Overall, delayed treatment with AjA effectively limits the progression of BLM-induced pulmonary fibrosis. It is noteworthy that the therapeutic AjA treatment seemed to be equally effective as compared to the “preventive treatment” started at the same day of BLM administration.

## Discussion

In this study, we assessed whether AjA, a synthetic cannabinoid, effective in preventing dermal collagen accumulation in a model of systemic sclerosis [16, 17] prevents and/or limits lung fibrosis induced by BLM treatment. Specifically, we first show that the preventive use of AjA (starting the same day of BLM instillation) counteracts lung fibrosis by reducing inflammation and thus myofibroblast differentiation and accumulation.

Myofibroblasts are metabolically active in producing extracellular matrix proteins and in particular interstitial collagens. It is widely accepted that myofibroblast differentiation, induced by TGF-β1 and characterized by *de novo* expression of α-SMA, is a key event in wound healing and in the pathogenesis of fibrosis.

Our in vivo experiments show that BLM-induced fibrosis is attenuated in mice treated with either 1 mg/Kg or 5 mg/Kg of AjA, given 1 h before BLM treatment and then once a day for the following 8, 14 and 21 days. Gene expression of products implicated in fibrogenesis (TGF-β1, α-SMA, pSMAD2/3, CTGF and PPAR-γ), as well as histological and biochemical hallmarks of fibrosis are profoundly modified by the preventive AjA administration.

It is well recognized that therapeutic interventions that interrupt the inflammatory phase are often protective in BLM-induced lung fibrosis [25]. Unfortunately, the same interventions are ineffective in human IPF. Therefore, it is important to assess the ability of any potential therapeutic agent to reduce lung fibrosis when delivered in the early fibrotic phase (i.e. 7/8 days after BLM delivery) [25, 26].

On this basis, more clinical relevant is the observation that AjA still effectively limits pulmonary fibrosis - by interfering with fibrogenic mechanism (s) and in particular by reducing myofibroblast accumulation and differentiation—when the treatment started at day 8 after BLM delivery. The beneficial effects we observed in this experiment are comparable to those observed in animals treated in the preventive manner. The reduction of collagen deposition, the morphometric scores of fibrosis and the changes in the expression of gene products related to fibrogenesis were similar to those observed in animals in which AjA treatment started at the same day of BLM administration.

Although we do not have conclusive explanations for these findings, it may be assumed that the therapeutic effects of AjA treatment both in *inflammatory* or in early *fibrogenic phase* are related to the nature (anti-inflammatory and anti-fibrotic) of this cannabinoid. For some time AjA has been mainly considered to exert anti-inflammatory activities [27]; however, recent data obtained in a model of systemic sclerosis suggest that AjA also posses anti-fibrotic properties [16, 17].

It has been recently shown that AjA directly and specifically binds to PPARγ, a transcription factor belonging to the nuclear hormone receptor family, that is widely expressed in immune cells and in many tissues (colon, spleen, liver, skeletal muscle, lung) [28, 29]. Very important findings obtained in different vitro studies demonstrated that AjA increases the transcriptional activity of PPARγ [30] that in turn prevents the translocation of NF-κB protein from the cytoplasm to the nucleus [31, 32], resulting in a reduced production of pro-inflammatory cytokines, metalloproteases and acute-phase proteins [30, 33].

Moreover, recent in vivo studies confirm that the modulation of PPAR-γ activity in the lung can influence inflammatory and immune response in endotoxemia [34] and in other pulmonary diseases, such as COPD and asthma [35].

PPAR-γ activation also plays important role in regulating processes related to fibrogenesis, including cellular differentiation and wound healing both in vitro and in vivo [29, 36]. Moreover, treatment with PPAR-γ ligands in a variety of mouse models ameliorated lung fibrosis [37–40].

Even though the molecular basis of the anti-fibrotic activity of AjA are far from clear, the recently reported relationship between the endocannabinoid system and PPAR-γ signalling pathway [41] suggests the possibility that AjA antifibrotic effects may be directly mediated through PPAR-γ action.

PPARγ synthetic ligands can interfere with TGF-β1 signaling in a SMAD-dependent way, thus counteracting the profibrotic effect of TGF-β1 [38, 42]. Consistent with these findings, we observed that after BLM administration the increased TGFβ1 expression and the activation of the TGF-β1 signalling pathway (as revealed by phosphorylated SMAD2/3) increased CTGF and α-SMA expression as well as the expression of collagen genes. The increased TGFβ1 expression was mirrored by a concomitant decrease of PPAR-γ expression.

As observed in the current study, AjA treatment both in *inflammatory* or in early *fibrogenic phase* does reduce the expression of TGFβ1 stimulating expression of the endogenous PPAR-γ and more interestingly its nuclear translocation. This may explain why also in the fibrogenic phase AjA can counteract the progression of fibrosis. Actually, PPAR-γ activators may oppose the fibrogenic effects of TGFβ1 by inhibiting fibroblast differentiation [29] or by modifying myofibroblast phenotype [43, 44].

The present study provides the first evidence on the efficacy of AjA as anti-fibrogenic agent in the lung. AjA can interfere with TGF-β1 pathway that leads to CTGF, α-SMA and COL1 and COL2 hyper-expression resulting in epithelial-mesenchymal transdifferentation and collagen deposition in lungs. The increase in the expression of PPAR-γ may also influence multiple regulatory pathways involved in fibrogenesis [38, 42]. Unfortunately, the mechanisms by which PPAR ligands alter fibrosis are not well understood, and additional work is necessary in this field.

Although BLM model does not completely mimic the clinical progression of IPF in patients [45, 46] and has significant limitations regarding the slow and irreversible progression of fibrosis, the BLM animal model is widely used for the assessment of potential anti-fibrotic agents. By using BLM model a large number of compounds have been shown to prevent fibrosis progression and have been suggested for clinical testing. However, to date none of these drugs have shown comparable success in IPF patients. One major issue is that most agents were given to the animals in a preventive regimen, prior to or simultaneous with BLM. In this setting, the drug action reflected more their anti-inflammatory activity than their capacity of influencing the subsequent events leading to fibrosis. When anti-fibrotic ability is considered, very few compounds resulted “true” anti-fibrotic agents if administered during the “fibrotic” phase of the BLM model.

## Conclusions

In conclusion, like very few other compounds AjA exerts potent anti-fibrotic effect when given during the fibrogenic phase of BLM lung. Further clinical studies are necessary to establish whether AjA may represent a new therapeutic option for treating human IPF, or other fibrotic lung diseases refractory to corticosteroids.
